# Lone Atrial Flutter in Children and Adolescents: Is It Really “Lone”?

**DOI:** 10.1007/s00246-020-02491-z

**Published:** 2020-11-09

**Authors:** Jana-K. Dieks, David Backhoff, Heike E. Schneider, Matthias J. Müller, Ulrich Krause, Thomas Paul

**Affiliations:** Department of Pediatric Cardiology and Intensive Care Medicine, University Medical Center, Georg-August-University Göttingen, Robert-Koch-Str. 40, 37075 Göttingen, Germany

**Keywords:** Atrial flutter, Catheter ablation, Endomyocardial biopsy, Radiofrequency current, Pediatrics, Adolescents

## Abstract

Atrial flutter (AFL) in children and adolescents beyond the neonatal period in the absence of any underlying myocardial disease (“lone AFL”) is rare and data is limited. Our study aims to present clinical and electrophysiological data of presumed “lone AFL” in pediatric patients and discuss the role of endomyocardial biopsy (EMB) and further follow-up. Since July 2005, eight consecutive patients at a median age of 12.7 (range 10.4–16.7) years presenting with presumed “lone AFL” after negative non-invasive diagnostic work-up had electrophysiological study (EPS) and induction of cavotricuspid isthmus (CTI) conduction block by radiofrequency (RF) current application. In 6/8 patients EMB could be taken. Induction of CTI conduction block was achieved in all patients. Histopathological examination of EMB from the right ventricular septum exhibited myocarditis or cardiomyopathy in 4/6 patients, respectively. During follow-up, 4/8 patients had recurrent arrhythmia (AFL *n* = 2, wide QRS complex tachycardia *n* = 1, monomorphic premature ventricular contractions *n* = 1) after the ablation procedure. 3/4 patients with recurrent arrhythmia had pathological EMB results. The remaining patient with recurrent arrhythmia had a negative EMB but was diagnosed with Brugada syndrome during further follow-up. Taking together results of EMB and further clinical course, only 3/8 patients finally turned out to have true “lone AFL”. Our study demonstrates that true “lone AFL” in children and adolescents is rare. EMB and clinical course revealed an underlying cardiac pathology in the majority of the individuals studied. EMB was very helpful in order to timely establish the diagnosis of myocarditis or cardiomyopathy.

## Introduction

In pediatric patients, atrial flutter (AFL) without any obvious cardiac abnormatility (“lone AFL”) is rare and most frequently occurs in the neonatal period [1]. Beyond this age, AFL primarily occurs in the presence of congenital heart disease (CHD) [1–3], in patients with accessory atrioventricular (AV) pathways [4], in myocarditis [5, 6] and in patients with various types of cardiomyopathy [7]. In a sentinel study from 1985 on AFL in the young with limited diagnostic methods available at that time, less then 10% of the patients had an “otherwise” normal heart (“lone AFL”) [8].

In adults, incidence of AFL increases with age [9]. Adult patients either have a history of ischaemic heart disease or prior cardiac surgery [10]. A familial risk has been described particularly in patients diagnosed before 50 years of age [11], and a genetic mutation predisposing for AFL has been identified [12].

In neonates and infants with AFL and a structurally normal heart, AFL is thought to be benign. After conversion into sinus rhythm, long-term antiarrthythmic therapy is not recommended as recurrences are very rare [1, 13]. In children and adolescents, depending on clinical presentation and efficacy of antiarrhythmic medication, electrophysiological study (EPS) and catheter ablation is the treatment of choice allowing for substrate specific treatment of AFL [14].

As actual data on AFL in pediatric patients beyond the neonatal period without any obvious underlying myocardial disease is limited, our study aims to present the clinical features and management of presumed “lone AFL” in this group of patients focussing on the value of endomyocardial biopsy (EMB) and the further clinical course.

## Patients and Methods

### Patients

Inclusion critera: All children and adolescents (< 18 years of age) referred to our tertiary pediatric electrophysiology center for EPS and catheter ablation of AFL in the absence of overt cardiac disease between July 2005 and January 2018 were studied. AFL was the only arrhythmia documented prior to EPS. Before admission to our institution, electrocardiogram (ECG) during sinus rhythm and transthoracic echocardiogram had been judged to be normal. After admission to our institution, all patients underwent a standardized non-invasive work-up (see below). Overt absence of cardiac disease was defined as follows: normal ECG [15], normal transthoracic echocardiogram according to standard criteria [16] (normal anatomy, cardiac dimensions appropriate for body weight, normal left ventricular fractional shortening/ejection fraction, normal right ventricular function and no pericardial effusion), and exclusion of any systemic disease with potential cardiac involvement. All patients had EPS and intended induction of cavotricuspid isthmus (CTI) block by RF application (see below).

### Non-invasive Work-Up

A detailed medical history including family history was taken. Age of AFL onset, clinical symptoms, number and duration of AFL episodes, and previous therapy were documented. A 12-lead surface ECG and a 24-h Holter were obtained prior to EPS. AFL on surface ECG was characterized and defined as typical (counterclockwise) or atypical (clockwise) according to standard ECG criteria [17]. All patients underwent detailed two-dimensional transthoracic echocardiographic evaluation to rule out structural cardiac abnormalities. Since 2009, contrast-enhanced cardiac magnetic resonance imaging (CMR) was performed including cine imaging, T1- and T2-weighted imaging, phase contrast flow measurements, volumetry, tissue characterization of the myocardium and late enhancement in three patients.

### Electrophysiological Study and Catheter Ablation

Written informed consent was obtained from all patients, their parent(s) and/or legal guardian(s) prior to EPS. All antiarrhythmic medication was withdrawn at least five half-lives prior to EPS if applicable. Following complete cardiac catheterization including hemodynamic assessment (oxymetry, manometry, and calculations of cardiac index and pulmonary vascular resistance) and right and left ventricular angiographies as well as selective coronary angiography, EPS, endocardial mapping and radiofrequency ablation (RFA) were performed according to standard protocols [18] using non-fluroscopic mapping systems [19, 20]. CTI block was induced by point-by-point RF applications using RF generator power settings of 30–50 W and a target temperature of 45 °C at the 4 mm tip of the irrigated 7F ablation catheter (Marinr MCXL, Medtronic, Minneapolis, MN, USA or CoolPath Duo, St. Jude Medical, St. Paul, MN, USA) for a maximum of 45 s per lesion. Primary endpoint of catheter ablation was termination of AFL during RF application where applicable, proof of bidirectional CTI conduction block according to standard criteria and non-inducibility of AFL after RFA. Bidirectional CTI conduction block was defined as an increase of the trans-isthmus conduction time by at least 50% in both directions when compared to pre-RF state [21]. All patients had programmed atrial stimulation including incremental and single, double and triple extrastimulus testing with a minimum cycle length of 200 ms prior to RFA ± following RFA. Before performing the post-ablation study, there was a waiting time of 30 min. Programmed ventricular stimulation ± isoprenalin provocation was performed in three patients prior to RFA.

### Endomyocardial Biopsy

Upon completion of EPS and RFA, five EMB from the right ventricular septum were taken in all patients in whom written informed consent could be obtained (*n* = 6) using a 7F Mullins Fast-Cath™ Sheeth (St. Jude Medical) and a 5.5F biopsy forceps (Cordis, Milpitas, CA, USA). Three EMB were fixed in buffered 4% formaldehyde solution (Carl Roth, Karlsruhe, Germany), and two EMB were stored in RNAlater® stabilization solution (Thermo Fisher Scientific, Waltham, MA USA) for evaluation of viral genoma. EMB were sent to the Department of Molecular Pathology, University Hospital Tübingen, Germany, for examination. Standard work-up [22] included immunohistopathology for signs of acute or persistent infection or inflammation, eosinophilia, primary or secondary cardiomyopathy and storage disease. Polymerase chain reaction examinations were performed from biopsy samples and blood to rule out myocardial or systemic viral infection.

### Follow-Up

After hospital discharge regular follow-up visits were scheduled at the referring pediatric cardiologist or at the outpatient clinic of our institution.

### Statistical Analysis

Data was handled with Microsoft Excel (version 2016, Redmond, WA, USA). Continuous variables are expressed as median (range) or mean ± standard deviation, categorical variables as percentages.

## Results

### Patients

A total of eight pediatric and adolescent patients meeting the inclusion criteria underwent diagnostic work-up and catheter ablation for presumed “lone AFL”. One patient was female. Median age at onset of AFL had been 12.7 (range 10.1–15.4) years. All patients had experienced symptoms during AFL (Table 1). 6/8 patients (75%) had suffered from sustained (≥ 30 s) AFL episodes. Of these patients, 2/6 patients had syncope related to the arrhythmia. Additional symptoms included palpitations, chest pain, dizziness and dyspnea. Prior to EPS, 3/8 patients had needed cardioversion for sustained AFL. The remaining 2/8 patients had non-sustained (< 30 s) AFL as documented on Holter monitor. 1/8 patients with high arrhythmia burden had been treated with antiarrhythmic medication. None of the patients had had a previous EPS.

**Table 1:** Clinical details

Variable	Patient 1	Patient 2	Patient 3	Patient 4	Patient 5	Patient 6	Patient 7	Patient 8
Patients’ demographics and symptoms								
Age at AFL-onset	10.1 years	14.9 years	13.5 years	11.2 years	10.8 years	11.9 years	15.4 years	13.9 years
Age at EPS	10.4 years	15 years	16.7 years	11.8 years	12.3 years	12.1 years	16.4 years	16.4 years
Frequency of AFL	5 times per month since 3 months	Recurrent episodes since a few months	Every 3 months since 3 years	Total of 3 episodes since 6 months	Every 3 months since 1.5 years	Recurrent episodes, sometimes daily, since a few months	Total of 2 episodes since 1 year	Total of 4 episodes since 2.5 years
Duration of AFL	Non-sustained	Non-sustained	Sustained	Sustained	Sustained	Sustained	Sustained	Sustained
Syncope	No	No	No	No	Yes	No	Yes	No
Other symptoms	Palpitations	Dizziness	Palpitations	Palpitations	Palpitations	Chest pain, dyspnea and dizziness	Dizziness	Palpitations, dyspnea, pressure on the chest
Non-invasive findings								
ECG in sinus rhythm prior to ablation	Incomplete RBBB (QRS duration 88 ms)	Incomplete RBBB (QRS duration 96 ms)	Normal	Normal	Normal	Normal	Normal	Normal
Holter monitors	Two episodes of non-sustained AFL	Normal	Normal	3 single SVES, 3 single PVC	Intermittent 2nd degree AV block	AFL, single PVC	Normal	Normal
Echocardiography	Normal	Normal	Normal	Normal	Normal	Normal	Normal	Normal
CMR	n.e	Normal	n.e	n.e	Normal	n.e	n.e	Normal
Invasive findings								
Hemodynamics and angiographies	Normal	Normal	Increased trabecularization of both ventricles, slightly reduced LV function, EF 51% (normal > 52%)	Normal	Normal	Elevated LVEDP (16 mHg; normal ≤ 12 mmHg)	Normal	Elevated LVEDP (13 mmHg; normal ≤ 12 mmHg)
Additional findings during EPS	None (AFL not inducible)	None (AFL not inducible)	Left AT, AF (degenerated from AFL)	AVNRT, AF (degenerated from AFL)	Additional FAT inducible once	FAT and AF (degenerated from AFL)	None	None
Results of endomyocardial biopsies	n.e	Minor fibrolipomatous changes consistent with early ARVC	Initial biopsy: normal repeat biopsy after 43 months: chronic lymphocytic myocarditis	Normal	Normal	Initial biopsy: chronic myocarditis, HHV6 persistence repeat biopsy after 16 months: result unchanged	n.e	State after myocarditis
Follow-up								
Duration of follow-up	18 months	16 months	47 months	31 months	117 months	75 months	24 months	12 months
Recurrence of arrhythmia	No	Yes, non-sustained wide QRS complex tachycardia; two weeks after EPS	Yes, monomorphic PVC (max 27% of all QRS complexes); 3 years after EPS	No	Yes, AFL; 5,7 years after EPS	Yes, 1^st^ recurrence AFL; one day after EPS; 2^nd^ recurrence MAT; early after 2^nd^ EPS	No	No
Repeat EPS	No, n.a	Yes, repeat induction of CTI conduction block; no sustained SVT and no VT inducible	Yes, ablation of left ventricular focus	No, n.a	Yes, two repeat procedures, closure of conduction gaps within the CTI	Yes, 1st repeat procedure: isolation of coronary sinus ostium, 2nd repeat procedure: RFA of MAT	No, n.a	No, n.a
Further clinical course	n.a	Propranolol for one year, genetic testing for CPVT, HCM and ARVC normal	Monomorphic PVC reduced after 2nd EPS and Bisoprolol, left ventricular dilatation DD tachycardia-induced DD DCM	n.a	After repeat EPS and death of brother beta blocker, ICD-therapy, genetic negative familial Brugada syndrome (unspecific heterozygous DSP-gene sequence variant)	Recurrences poorly controlled with antiarrhythmic medication, free from tachycardia without any treatment after 3rd EPS	n.a	n.a
Final diagnoses	“Lone AFL”	Not established	Chronic lymphocytic myocarditis	“Lone AFL”	Familial Brugada syndrome	Chronic myocarditis with HHV6 persistence	“Lone AFL”	State after myocarditis
Status at end of follow-up	Free from tachycardia	On propranolol free from tachycardia, medication stopped on last follow-up visit	On bisoprolol reduced PVC (254/24 h)	Free from tachycardia	On sotalol free from tachycardia, ICD	Free from tachycardia	Free from tachycardia	Free from tachycardia

*AF* atrial fibrillation, *AFL* atrial flutter, *ARVC* arrhythmogenic right ventricular cardiomyopathy, *AT* atrial tachycardia, *AV* atrioventricular, *AVNRT* atriventricular nodal reentrant tachycardia, *CI* cardiac index, *CMR* cardiac magnetic resonance imaging, *CTI* cavotricuspid isthmus, *CPVT* catecholaminergic polymorphic ventricular tachycardia, *DCM* dilated cardiomyopathy, *DSP* desmoplakin, *EF* ejection fraction, *EPS* electrophysiological study, *FAT* focal atrial tachycardia, *HCM* hypertrophic cardiomyopathy, *HHV* human herpes virus, *ICD* implantable cardioverter defibrillator, *LVEDP* left ventricular enddiastolic pressure, *MAT* multifocal atrial tachycardia, *n.a.* not applicable, *n.e.* not examined, *PVC* premature ventricular contractions, *RBBB* right bundle branch block, *SVES* supraventricular extrasystoles, *SVT* supraventricular tachycardia, *VT* ventricular tachycardia

### Non-invasive Findings

Median age at diagnostic work-up in our institution was 13.6 (10.4–16.7) years, body weight was 54 (33–100) kg. Median interval between AFL onset and EPS was 10 (1–38) months.

#### Electrocardiogram

ECG prior to EPS showed incomplete right bundle branch block in 2/8 patients (25%). All patients had conduction times and electrical forces within age-appropriate limits. None of them had evidence for repolarization abnormalities (Table 1).

In all patients, counterclockwise AFL with a median cycle length of 236 (190–280) ms and variable AV conduction (1:1 to 4:1) had been documented.

#### Holter Monitoring

24-h Holter monitoring revealed normal sinus rhythm in 6/8 patients (75%). Of the remaining two patients, one was in sustained AFL and the other patient had two episodes of non-sustained AFL documented.

#### Transthoracic Echocardiography

Echocardiographic work-up revealed normal results in all patients.

#### Cardiac Magnetic Resonance Imaging

3/8 patients had CMR with normal results in all of them.

### Invasive Findings

#### Hemodynamic Studies

Complete invasive hemodynamics and angiographies for evaluation was available in all patients. Hemodynamics were completely normal in 6/8 patients (75%; Table 1). The remaining two patients had an elevated left ventricular end diastolic pressure (LVEDP of 13 and 16 mmHg, respectively). On angiography, one patient exhibited increased trabecularization of both ventricles and a slightly reduced left ventricular function with an ejection fraction (EF) of 51% (reference values in male patients: normal 52–72%, slightly reduced 41–51%, reduced 30–40%, and markedly reduced < 30%) [23].

#### Electrophysiological Study: Characterization of Atrial Flutter and Associated Arrhythmias

AFL was inducible by programmed stimulation in 6/8 patients (75%). CTI-dependent AFL was verified by positive entrainment within the CTI in all of them. During EP assessment, spontaneous degeneration of AFL into atrial fibrillation was observed in 3/8 patients. In the remaining two patients (25%), AFL was not inducible during EPS.

A median of 15 (9–42) RF applications with a median total burning time of 10.3 (4.7–24.6) min was required for induction of CTI conduction block. At repeat programmed atrial stimulation after RFA, one patient had inducible AV nodal reentrant tachycardia. RFA of the slow conducting pathway was performed during the same procedure. Three patients had unifocal (*n* = 2) or multifocal atrial tachycardia (*n* = 1) which were not targeted during the intial procedure because these tachycardias could not reproducibly be induced or were only short-lasting.

Median total procedure time was 192 (142–230) min, median total fluoroscopy time was 12.3 (2.7–27.2) min including hemodynamic assessement and angiography as well as obtaining EMB.

#### Acute Success and Complications

In the two patients lacking induction of AFL during EPS, induction of bidirectional CTI conduction block was empirically performed. Termination of AFL during AFL was achieved in the remaining six individuals. All eight patients had a successful ablation procedure as defined above. No procedure-related complications were observed.

#### Endomyocardial Biopsy and Histopathological Findings

EMB could be taken in 6/8 patients. In 3/6 patients (50%), EMB revealed pathologic findings. One patient had evidence of chronic myocarditis with HHV6 persistence, another patient had persisting myocarditis, and one patient showed minor fibrolipomatous changes consistent with an early state of arrhythmogenic right ventricular cardiomyopathy (ARVC). In the remaining three patients EMB were considered normal.

During further clinical course 2/6 patients had repeat EMB taken. In the patient with chronic myocarditis, repeat analysis showed unchanged results. In one patient with initially normal EMB results, there was evidence for chronic lymphocytic myocarditis during follow-up.

Taking initial and repeat EMB together, 4/6 patients (66.6%) had pathological results. Detailed information on results of EMB is provided in the Table 1.

#### Follow-Up

During a mean follow-up of 43 ± 31 months, cardiac arrhythmia events defined as any event of supraventricular or ventricular tachyarrhythmia were registered in 4/8 patients early (within 4 weeks; *n* = 3) or late (68 months; *n* = 1) after the procedure, respectively. Recurrent AFL was documented in 2/4 patients, broad QRS complex tachycardia in one, and monomorphic premature ventricular contractions (PVC) in another patient. Of these four patients, three had pathological EMB results, the remaining patient was diagnosed with familial Brugada syndrome six years after AFL ablation.

All four patients with cardiac arrhythmia events during follow-up had repeat EP procedures. Two individuals had repeat successful induction of CTI conduction block after four weeks and 68 months, respectively. One patient had RFA of a left-ventricular ectopic focus after 43 months. The remaining patient needed two EPS/ablation procedures after five and 21 months for multiple focal atrial tachycardia while complete CTI conduction block was proven.

On their last follow-up visit, 6/8 patients (75%) were without any evidence of tachycardia without any specific treatment. One patient still had PVC (250/day) four years after the initial RFA while taking bisoprolol 5 mg bid. In the last patient diagnosed with Brugada syndrome, control of breakthrough atrial tachycardias was achieved with sotalol 80 mg bid.

The patient whose EMB had shown minor fibrolipomatous changes consistent with early ARVC developed non-sustained wide QRS complex tachycardia early after EPS. Repeat EPS did not show a substrate for this recurrent arrhythmia. Repeat CTI conduction block was induced to close a residual conduction gap. Consecutively, the patient was started on propranolol. He had negative genetic testing for catecholaminergic polymorphic ventricular tachycardia, hypertrophic cardiomyopathy and ARVC. After 16 months of follow-up, he was free from tachycardia while on antiarrhythmic medication.

At the conclusion of follow-up of our patient cohort, only 3/8 patients met the definition of true “lone AFL”.

## Discussion

Over the last 14 years, a limited number of patients had been referred to our tertiary pediatric electrophysiology center for EPS and ablation of presumed “lone AFL” suggesting a very low incidence of the tachycardia in this age group. One main finding of the present study is, that “lone AFL” might actually not be “lone” at all in this age group, as after negative non-invasive workup, previously un-diagnosed underlying pathologies were present in 4/6 biopsy specimen (66.6%). Moreover, in one patient with recurrent arrhythmia and negative EMB, familial Brugada syndrome was diagnosed later-on.

### Epidemiology of “Lone Atrial Flutter”

Data regarding the prevalence of AFL in patients with a structurally normal heart is limited. In an initial study from 1985, 6.3% of 380 young AFL patients aged one to 25 years had a normal heart defined as no repaired, palliated or unoperated congenital heart disease, no cardiomyopathy, no rheumatic heart disease and no other lesions. However, in the early 80ies of the last century, diagnosis of a “normal heart without evidence of cardiomyopathy” had been established by physical examination, chest X-ray, occasionally 2D-echocardiography (35.2% of all patients) and mainly by cardiac catheterization (67.4% of all patients). EMB and MRI had not been performed at all [8].

During the period of our study, a total of 870 pediatric patients underwent catheter ablation for SVT or atrial tachycardia at our institution. Taking this group of patients as denominator, incidence of presumed “lone AFL” was 0.9%, while incidence of “true” lone AFL was 0.3%. It is of note that in five of our eight patients initially assessed to have “lone AFL”, EMB and the mid-term clinical course revealed significant pathological findings including additional supraventricular or ventricular tachycardias, cardiomyopathy, channelopathy or myocarditis (*n* = 3). For myocarditis, the mechanism of arrhythmogenesis has been described to be the interaction of host (inflammation) and viral (altered cellular signaling) factors that can induce ion electrophysiological and structural remodeling leading to triggered and reentrant arrhythmia [24]. Especially for acute myocarditis, several outcome predictors have been described focusing on arrhythmia burden and reduced ventricular function mainly during the first hospitalization. Here, in the early phase, arrhythmias were related with a worse outcome [6]. However, after discharge from hospital, a majority of patients did not require continued antiarrhythmic treatment and recurrences are rare [25]. In our study, of the three patients with myocarditis, one required repeat EPS and additional long-term antiarrhythmic treatment and one required two repeat EPS. All three patients were free from rachycardia at the end of follow-up.

### Underlying Cardiac Pathology and Impact of Endomyocardial Biopsy

In a significant number of our study patients, transient or persistent underlying cardiac pathologies were identified. EMB substantially contributed to diagnosis and individual management. However, taking the probability of false negative biopsy results due to a sampling error and that permission for EMB was denied in two patients, the number of patients with true “lone AFL” may have even been lower than assessed. However, those two patients who had no EMB taken were free from cardiac arrhythmia events during a follow-up of 18 and 24 months, respectively.

EMB was safe in our group of patients and contributed significantly to establishing the final diagnosis. Severe complications in children, however, have been reported [26].

### General Considerations

In the present study, acute success of AFL ablation by induction of CTI conduction block was achieved in all our patients during initial EPS which is consistent with the high efficacy > 90% as reported for AFL ablation in adults [27–30]. In adults, half of atrial arrhythmia recurrences following AFL ablation were documented early within the first month after EPS [29] with atrial fibrillation being a significant co-finding with an incidence of approximately 30% [27–29]. In our study group, AFL recurrences predominantly developed within the first weeks after ablation raising the question whether these events have to be judged as failures instead of early recurrences.

Only a limited number of our study patients had CMR studies. Interestingly, in our experience, CMR did not add any significant information to the diagnostic work-up. The number of patients, however, was extremely limited. No final conclusion on the impact of cardiac MRI in this setting can be drawn.

It also needs to be considered to include further genetic testing in the early standard work-up of presumed lone AFL in children and adolescents as it is already routine in many centers around the world. This recommendation is underlined by our finding, that at least one of our study patients was found to have a familial cardiomyopathy.

## Conclusions

In a significant number of patients with presumed lone AFL, EMB revealed evidence for underlying cardiac disease. Therefore, detailed non-invasive and invasive diagnostic work-up including EMB is recommended in young patients with presumed “lone AFL” allowing disease-specific treatment. The role of CMR and genetic testing in this setting remained undetermined but its use is strongly recommended in order to obtain relevant data in the future.

## Limitations of the Study

Limitations of our study include the retrospective design and the single-center approach. Only a small number of patients fulfilled criteria of presumed lone AFL. As data in the literature is sparse, we have the feeling that our results on this entity are of major impact and may serve as guidance for systematic work-up of future young patients with presumed “lone AFL”. As the role of CMR is still undefined, cost-effectiveness also needs to be discussed.
